# Genetics and genomic medicine in Iran

**DOI:** 10.1002/mgg3.606

**Published:** 2019-02-27

**Authors:** Babak Behnam, Maryam Zakeri

**Affiliations:** ^1^ Department of Medical Genetics and Molecular Biology College of Medicine Iran University of Medical Sciences (IUMS) Tehran Iran; ^2^ Non‐Communicable Disease (NCD) Group Department of Health Hormozgan University of Medical Sciences Bandarabbas Iran

## Abstract

Attention has been focused on the field of genetics and genomics in Iran in recent years and some efforts have been enforced and implemented. However, they are totally not adequate, considering the advances in medical genetics and genomics in the past two decades around the world. Overall, considering the lack of medical genetics residency programs in the Iranian health education system, big demand due to high consanguinity and intraethnic marriages, there is a lag in genetic services and necessity to an immediate response to fill this big gap in Iran. As clarified in the National constitution fundamental law and re‐emphasized in the 6th National Development Plan, the Iranian government authority is in charge of providing the standard level of health including genetic services to all Iranian individuals who are in need.

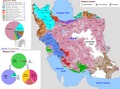

## 1 BACKGROUND

Iran (Persia; officially the Islamic Republic of Iran), is the 18th most populous country (with over 81 million individuals) in the world (www.amar.org.ir). Iran is located in Western Asia with 1,648,195 km^2^ land, as the second largest country in the Middle East and the 17th in the world. Iran is bordered to the north by the Caspian Sea, to the south by the Persian Gulf and the Gulf of Oman, to the northwest by Turkey and Armenia and the Republic of Azerbaijan, to the west by Iraq, to the northeast by Turkmenistan, and to the east by Afghanistan and Pakistan. Tehran is the country's capital, its largest and the leading economic and cultural city.

**Figure 1:**
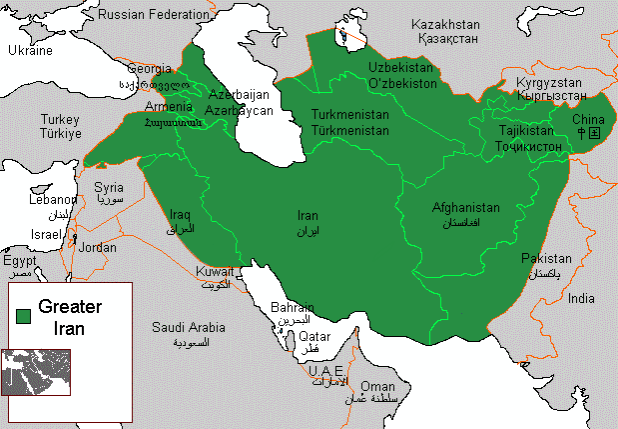
Greater Iran in terms of ethnicity from historic point of view (By Hosseiniran ‐ paint, CC0, https://commons.wikimedia.org/w/index.php?curxml:id=42801778)

Iran is the origin of the world's oldest civilizations (Barrington, 2012; Whatley, 2001) since Elamite kingdoms in the fourth millennium BCE to Iranian Medes in the seventh century BCE when it was first unified (Encyclopædia Britannica, 2010); as the largest empires in history from Eastern Europe to the Indus Valley (Achaemenid) was founded in Persia in the sixth century BCE by Cyrus the Great (Sacks, Murray, & Brody, 2005). Greater Iran used to refer to the regions with Iranian culture including the Caucasus, and parts of West, South, and Central Asia (https://en.wikipedia.org/wiki/Greater_Iran) (Figure 1). This extensive Iranian culture in other regions could be due to a long life contact with the various imperial dynasties of Iran, whereas North Caucasus was not under straight Iranian rule. It also could be either due to being part of Persian Empire (e.g., Medes, Achaemenids, Parthians, Sassanians, Samanids, Safavids, Afsharids, and the Qajars) for a long historical period of time; or happened by so many peoples who promote and respect their cultures (e.g., Bahrain, Tajikistan, and the western parts of South Asia).

In the fourth century BCE, it fell to Alexander the Great leading to division into some Hellenistic states. An Iranian disobedience developed to the Parthian Empire followed (in the third century CE) by the Sasanian Empire as a leading world power till the seventh century CE when Arab Muslims conquered it and displaced the Zoroastrianism and Manichaeism with Islam (Jeffreys & Haarer, 2006; Stillman, 1979).

Iran made major contributions with so much impact on art, science, and medicine for some centuries, including *Avicenna* in the 10th century who authored *The Canon of Medicine* (*Al‐Qanun fi't‐Tibb)*. *The Canon of Medicine* was a five‐volume medical encyclopedia used as the reference medical textbook in Europe up to the 18th century (McGinnis, 2010). Then Iran was conquered by the Mongols and the Turks till an Iranian state and nationality reestablished in the 15th century (Curtis & Stewart, 2005). In the 18th century, Iran was one of the most powerful countries; however, a series of conflicts with the north neighbor Russian Empire led to significant territorial losses in the 19th century (Dowling, 2014; Fisher et al., 1991), followed by the 1953 coup and 1979 revolution.

**Figure 2:**
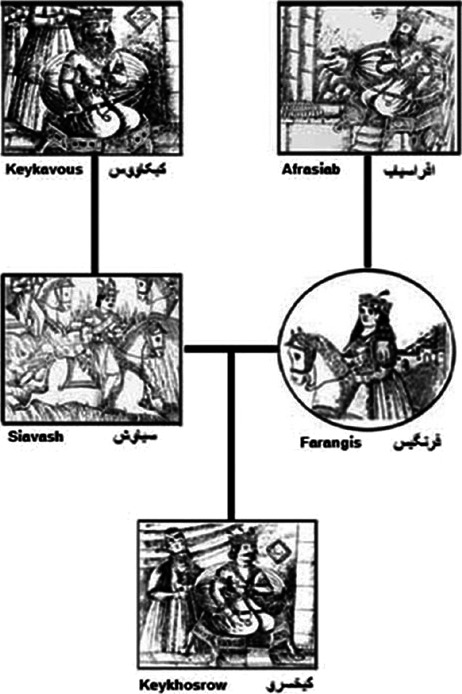
Flowchart showing Keykhosrow's inheritance of characteristics from both parents’ (mother and father) fathers (Keykavous & Afrasiab) (by Kariminejad MH & Khorshidian A. Indian J Hum Genet. 2012)

Interestingly, prior and consistent with what has been currently proved and accepted as a fact, Persian historically believed in the equal contribution of women and men in formation of a child and its hereditary characteristics since the prophet Zoroaster (~1,700 years BC) who expressed equal right for both sexes to choose how to live life. To store the egg (gametes) from reproductive organs (gonads) for future conception was also mentioned in *Vandidad* book (by *Mades* in the *Median* dynasty; 800–700 BC). Also 800 years ago, “the Great Iranian Poet” Ferdowsi of 10th century explains an equal contribution of man and woman in the transmission of characters and fetus formation; it is mentioned in *Shahnameh*—the world's longest epic poem created by a single poet, and the national epic of Greater Iran (Browne, 1998). Regarding an Iranian prince—*Keykhosrow*‐, Ferdowsi versified “this handsome child has got the royal characteristics of two kingdoms: from *Afrasiab* (the king of Turan) and *Keykavus* (the emperor of Iran)” (Abolqasem, 2007), respectively, his maternal and paternal grandfathers. This is directly addressing to both sides’ lines of descent and the traits inheritance from ancestors, whereas the maternal ancestor is mentioned prior to the paternal one (Figure 2) (Kariminejad & Khorshidian, 2012). However, world scientists did not believe in reproduction (but believed in *ovism* and other hypotheses) till renaissance happened. In the recent 2–3 centuries tremendous efforts have been made in the field of reproduction biology including the embryo formation via ovum and spermatozoa union (fertilization).

**Figure 3:**
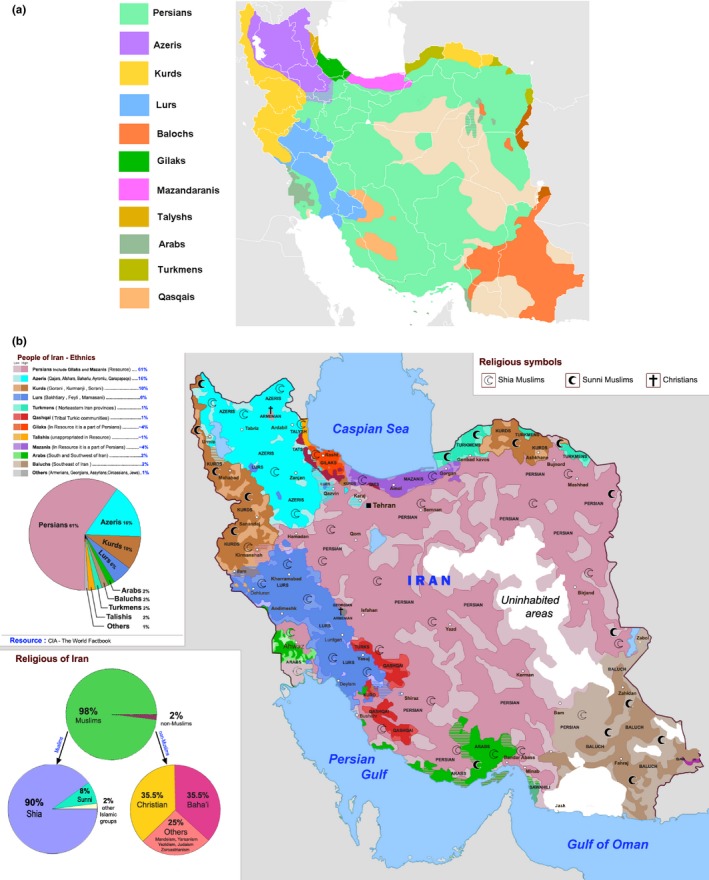
(a) Multi‐ethnic Iran (By Hosseiniran at English Wikipedia, CC BY‐SA 4.0, https://commons.wikimedia.org/w/index.php?curxml:id=50447706). (b) Iranian Ethno‐language‐religious map (By Worldmaper ‐ Own work, CC BY‐SA 4.0, https://commons.wikimedia.org/w/index.php?curxml:id=41510939)

From population demography viewpoint, Iran is a big regional multicultural country consisting of numerous ethnic and linguistic groups, including Persians (61%; including Gilaks and Mazandaranis 4% each), Azeris (16%), Kurds (10%), Lurs (6%), Balochs (2%), Arabs (2%), Turkmens (1%), Talyshs (1%), Qashqais (1%) (The World Factbook – Iran, [Link]). Iran population from religious point of view, 99.39% are Muslims including Shia (90%–95%), Sunni (4%–8%), and other Islamic subgroups (2%) (Figure 3a–b) (https://en.wikipedia.org/wiki/Ethnicities_in_Iran).

The female and male median age of the Iranian population is 31.3 and 30.9 years (totally 31.1 years), respectively, with 75.1% living in urban areas. The biggest metropolitan areas are the cities of Tehran (13.26 million), Mashad (3.37 million), Isfahan (2.24 million), Shiraz (1.87 million), Karaj (1.85 million), and Tabriz (1.77 million).

Consanguineous marriages are allowed in Iran, and are common in most ethnic groups, communities, and religions of Iranians including Azeris, Kurds, Lurs, Balochs, Arabs, Turkmens, Gilaks, Talyshs, Qasqais, and Mazanis. Consanguineous marriage is not only common but also has a positive trend in Iranian community. In a survey in Persian population of Iran, the consanguinity status (rate) for 1,789 marriages showed 8.8% in the generation with marriages before 1949 versus 19% after 1979 (Akrami, Montazeri, Shomali, Heshmat, & Larijani, 2009). Moreover in the recent years, the consanguineous marriage rate has been reported as high as 38.6% with the highest among first cousins (27.9%) (Saadat, Ansari‐Lari, & Farhud, 2004).

According to the reports by the Ministry of Health and Medical Education (MHME) in Iran, the mean maternal and paternal ages are 28 and 33 years, respectively; and the total fertility rate is 2.01 children born/woman. The current birth and growth rates are, respectively, 10.6 births per 1,000 inhabitants and 1.24. Changes in socioeconomic status (SES) may contribute to the increasing parental age and low fertility rates. Higher education levels of women and their more exposure to the workforce vacancies from one way, and gender inequality, the consequences of sanctions and economic hardship from another way, play an important role in above‐mentioned facts and rates. All these resulted in a significant increase in the age of marriage and maternal age above 35 years old, which may have a direct increasing effect on congenital and genetic defect in subsequent generations (Heidari & Dastgiri, 2014). Also there is an active process of immigration from Iran which have a considerable impact on the ethnic diversity via genetic drift and shuffling mainly because of different SES. There is a trend of improvement in pregnancy care (i.e., routine folic acid supplementation), and increase in the general healthcare of the population. Also based on the religious permission (*Fatwa*) and because of prenatal screenings and public education, there is a significant increase in the voluntary termination of pregnancies upon the detection of fetal abnormalities. The frequency of congenital disorders is notably high (38.3/1,000) in Iran, including congenital malformations (17.9/1,000), single gene disorders (16.4/1,000 with G6PDd), chromosomal disorders (3.1/1,000), and congenital infections (0.9/1,000) (http://ghdx.healthdata.org/organizations/ministry-health-and-medical-education-iran). The greatest prevalence and proportion rates belonged to musculoskeletal disorders, skin, and urogenital anomalies, and may be different in different ethnicities due to higher consanguineous marriages, different genetic makeup and SES (Vatankhah, Jalilvand, Sarkhosh, Azarmi, & Mohseni, 2017). A mild decrease has been detected just in the frequency of anencephaly and spina bifida since 1990s when folate was added as a routine supplement for the women in reproductive age prior to pregnancy and in the first trimester, in Iran.

## 2 METHODS

### 2.1 Legal, social, and ethical implications of genetic testing in Iran

The Office of Study for Humanistic and Islamic Science on Medicine and Medical Ethics at the MHME started its activity in 2001 and has drafted the 27‐clause act entitled, “The Protection Code for Human Subjects in Medical Research” (Iranian National Commission for UNESCO, 2001). The most important ethical issues included: informed consent, human rights during research, certifying research projects according to risks and benefits, privacy of information, paying compensation for harm imposed by research on human subjects, cultural and religious implications of research, rights of prisoners, and individuals with mental retardation and of psychotic patients, and research on the fetus. The Act has been customized according to the Code of Religious Laws and cultural issues peculiar to the Iranian population. Then, MHME required all universities and biomedical research centers to develop bioethics committees based on a uniform guideline which was prepared by the Office of the Deputy for Research, MHME. The parliament of the (Islamic Republic of) Iran approved an act on abortion in 2005, and under this law, a pregnancy could be terminated within the first 4 months of pregnancy (18 weeks by LMP), if the fetus was mentally or physically handicapped, or where the mother's life was likely to be in danger; as long as three specialists confirmed the problem (IR Iran Parliament, 2005).

### 2.2 Prenatal diagnosis and therapeutic termination of pregnancy

Prenatal diagnosis (PND) is an acceptable practice in Iran. There are two main legal grounds for PND in Iran; the main one is the endangerment of a mother's life, and the other one is parents’ severe hardship. The “hard to treat diseases” include chromosomal disorders, major hemoglobinopathies, inborn errors of metabolism, Duchene muscular dystrophy, and spinal muscular dystrophy which are all diagnosed early in embryonic period that medical abortion is possible and advisable in Iran The Iranian law, based on religious authorities (fatwa), allows for termination of pregnancy before the 19th week of embryo (the 120th day of the pregnancy by LMP) on the affected fetus with a severe disease in ultrasonography imaging and/or by a detected disease‐causing mutation/chromosomal aberration including fetuses with Down syndrome. Most of the population of Iran are religious/traditional so the interruptions of pregnancy were relatively rare. However, *fatwa* has put forth in the recent years, that “any” fetal disease may be terminated if it puts the parents in hardship (Kosaryan & Rabiei, 2014). Preimplantation genetic diagnosis (PGD) is provided in private or semi‐private sectors in very limited centers. PGD is recommended mainly by the obstetricians and gynecologists (OB/G) specialists for couples at risk for the chromosomal aberrations and very well‐known disease‐causing mutations.

Despite this, extensive changes in the healthcare system including ethical changes are still necessary to overcome the ethical obstacles including knowledge gap and informed consent, privacy and confidentiality and availability of healthcare services. The approaches to genetic testing are limited but diverse in Iranian, mainly because of the differences in the traditions of different ethnicities. The multi‐ethnic Iranian communities (composed of more than 10 ethnic groups) are homogeneous, and include about 50% of Iranian population.

### 2.3 The genetic law in Iran?

The purpose of the law is to provide the screening tests, genetic testing, and genetic counseling for early detection of some targeted genetic diseases to all, including women in reproductive ages, and pregnant women. It is defined in section 75 of the 6th National Development Plan (2016) (https://shenasname.ir/1391-09-30-20-01-30/tosee/plan6/3579.html), the Ministry of Health and Medical education (MHME) is in charge to recruit cooperative and private sectors to provide accredited genetic laboratories to the couples who are in need. Also MHME and State Welfare Organization of Iran (http://en.behzisti.ir/Portal/Home/) need to provide the access to genetic counseling to them. There is a possibility to perform genetic tests outside the country if necessary.

### 2.4 Medical/Human genetic professionals in Iran

As mentioned above, despite all other residency programs in different disciplines, medical genetics has not been yet a medical specialty recognized by MHME and the Iranian Medical Council (IRIMC) (http://irimc.org/). However, MHME defined the curriculum and professional criteria for Ph.D. in medical genetics in five universities, with the annual acceptance of 15 holders of a master degree (in human genetics, biomedical sciences, and midwifery). The Ph.D. in medical genetics program includes 24‐month syllabus of cytogenetics, molecular, biochemical, and medical/clinical genetics followed by a comprehensive exam. After passing the comprehensive exam, doing a dissertation and oral (defense) examination is necessary prior to taking the medical genetic national board examination. The board‐certified Ph.D. medical geneticists are recognized as laboratory specialties in the health system by MHME and IRIMC. There is no separate training program for cytogenetics, molecular, and biochemical genetics specialties. A Ph.D. medical geneticist can serve as director of a genetics laboratory, and sign out the results of tests in the fields of cytogenetics, molecular, and biochemical genetics.

Also a fellowship in molecular pathology after completion of pathology residency program in an approved pathology department is available for pathologists. There is an accredited 2‐year Master degree training program in human genetics in 16 Iranian universities; 82 students have been accepted in the M.Sc. human genetics in the current academic year. One with the M.Sc. in human genetics can serve as a genetics laboratory manager; and every genetics laboratory requires to have at least a manager with Master degree in human genetics. There is no particular training program for the genetic counseling.

### 2.5 Budgeting of genetic services in Iran

The total budget of Iran's healthcare system is 5.95 Billion USD, which represents 6.9% of the GDP, whereas 4% comes from private resources. Although the overall health expenditure index has been raised 71 times during the past 20 years, it is still very low per capita in Iran with, respectively, 47.8% and 41.2% out‐ of‐ pocket and public health expenditure (Khosravi, Soltani, Javan‐Noughabi, & Faramarzi, 2017). Iranian government (MHME) funds 42% of the budget, and support the major burden in the public funding of healthcare.

Genetic tests for screened cases or the ones whom recommended by a physician because of suspicions of any chromosomal aberration, thalassemia, and/or PKU are covered by insurances. Also the health insurances mostly cover the costs of molecular genetic tests as long as their indications are well‐defined (because of being well‐known disease‐causing mutations) and they are carried out in Iran. Two examples for indicated well‐defined mutations could be p.508del mutation in *CFTR* (cystic fibrosis), and 35delG in conexin26 (deafness). Meanwhile, the tests are mostly affordable for people in need in above‐mentioned cases. The costs of IVF and the genetic services which are necessary because of infertility are also covered by the national health insurance. Otherwise, many genetic tests are not covered by health insurances and therefore are hardly affordable for most of the families in need.

### 2.6 Clinical genetic laboratories

By now, there are 49 clinical genetic (diagnostic) laboratories in different provinces of Iran; including 13 in Tehran (Tehran), 5 in Fars (Shiraz), 4 in Isfahan (Isfahan), 2 in Azarbayejan (Tabriz), 4 in Khorasan (Mashad), 4 in Khoozestan (Ahvaz), 1 in Balochestan (Zahedan), 3 in Kerman (Kerman), 1 in Kermanshah (Kermanshah), 3 in Gilan (Rasht), 4 in Mazandaran (3 in Sari & 1 in Babol), 2 in Hormozgan (Bandarabbas), 1 in Hamedan (Hamedan), 1 in Kurdestan (Sanandaj), and 1 in Alborz (Karaj). About half of them are in charge of the national screening program for assigned genetic disorders (thalassemia, PKU, etc).

Almost all genetic laboratories in Iran have both the cytogenetic and molecular genetics divisions; the set‐up of each genetic laboratory is individual and different from others. The directors of both cytogenetics and molecular genetics divisions are Ph.D. in medical genetics. S/he could be director of both simultaneously. The cytogenetics laboratories provide two types of services: clinical diagnosis and PND. However, the cytogenetics laboratories are independent and found outside of the genetic departments in hospitals, as there is no such clinical department/unit due to the lack of medical genetics program as a clinical discipline.

All the genetics laboratories are regulated by the MHME. There are just three active laboratories offering Chromosomal microarray analysis (CMA) for clinical and PND in Tehran. Perhaps there are three more laboratories which offer CMA in three big cities (Isfahan, Ahvaz, and Shiraz) which are mainly for PGD and PND. Cytogenetic tests of oncology/hematology are also performed in the genetics laboratories or in the oncology/hematology departments (Bone Marrow Transplantation in a hospital affiliated with Tehran University of Medical Sciences, TUMS). Generally, the genetic tests for diseases which are specific to a hospital and/or department (e.g., ophthalmology, endocrinology, oncology/hematology, etc) are set up in a very limited number of genetic laboratories within the hospitals.

The number of genetic laboratories which are licensed and regulated by the MHME are growing rapidly in the recent years. However, there are (for maximum) just 4–5 big and mother private genetic laboratories in capital Tehran providing cytogenetics and molecular genetic testing.

The performance of next‐generation sequencing (NGS) was literally announced in 2016 in Iran. However, the samples for different diagnostic panels (WES and GWAS) are still shipped outside the country, rather than being performed as a clinical service in any of the Iranian accredited laboratories. In turn, it is due to lack of the NGS platforms hardware maintenance and support (by the main company providers) for the analysis of genetic variation secondary to Iranian Transactions and Sanctions Regulations (ITSR).

## 3 RESULTS

### 3.1 The Iranian national genetic database (Iranome)—integrating research and clinical genetics

Iran with its very diverse population in terms of ethnicity, is among the most underrepresented populations in currently available human genomic variation databases; this is important because many genomic variations are ethnicity‐specific.

Similar to the “100,000 Genomes Project” funded by the Department of Health of the United Kingdom (http://www.genomicsengland.co.uk), the Iranian MHME with its partner—Genetics Research Center (GRC) at the University of Social Welfare & Rehabilitation Sciences, Tehran, Iran and Dalla Lana School of Public Health at University of Toronto, Toronto, Ontario, Canada—have recently (2017) decided to launch the Iranome database (available at www.iranome.com). It has been launched by performing whole‐exome sequencing on 800 individuals from eight major ethnic groups in Iran. The groups included 100 healthy individuals from each of the following ethnic groups: Arabs, Azeris, Balochs, Kurds, Lurs, Persians, Persian Gulf Islanders, and Turkmen. They represent over 80 million Iranians, and perhaps over half a billion populations in Middle East with a rapid population growth expectations for the future (MENA Policy Brief, 2001) Despite the shortages in clinical genetic services in Iran, there is a significant increase in the number and quality of publications in this field in the past two decades.

### 3.2 Genetic services in Iran

#### 3.2.1 Genetics and genetic counseling in the hospitals and community

No work was reported on genetics in Iran until 1936 when a genetic course was added to the curriculum of biology in the universities. The Iranian Genetics Society was founded in 1966. The first‐genetic counseling clinic opened, and the first department of Human Genetics and Anthropology was founded in 1972 at the School of Public Health, University of Tehran by DD Farhud. In 1986, he reported some genetic information on the Persian population including higher rates of chromosomal, external genital, and thorax and abdominal anomalies, and other syndromes; similar rates of cleft lip and palate, joint dislocation, and finger anomalies in Iranian infants compared to other populations were found (Akbari, Papiha, Roberts, & Farhud, 1986; Farhud, Walizadeh, & Kamali, 1986). The first diagnostic (cyto)‐genetic laboratory was established in 1979 by MH Kariminejad (http://www.irangenepath.com/).

Although medical genetics have been practiced by a number of physicians and/or scientists for several years in Iran, there is still no medical genetics and clinical genetics residency and fellowship program accredited by MHME.

Now, the departments of medical genetics have been established in several medical schools. However, medical genetic services in Iran are not well‐organized in a uniform, comprehensive, and academic discipline mainly due to the lack of residency program in medical and clinical genetics. There is no accredited degree for genetic counseling too. Some of the units are offering genetic services in independent units with different sizes in some university hospitals. The services which are offered in these units/departments mainly include genetic counseling, cytogenetic services with the ability of prenatal diagnosis for the common chromosomal aberrations, and a very limited number of molecular genetic tests (beta‐thalassemia, panel of thrombophilia, etc). The above‐mentioned genetic services and tests in the medical genetics laboratories of the university hospitals are offered to the public and covered by the medical insurances. In all these university hospital medical genetic laboratories, there is at least one PhD (or rarely MD/PhD) in medical genetics graduated from a foreign university (and qualified after the review and evaluation by the Iranian board of medical genetics as PhD in Human/Medical Genetics). Otherwise, they should have graduated from a PhD program in Medical Genetics from a local university (within Iran). There are just 2–3 MHME‐accredited programs of PhD in Medical Genetics. The genetic counseling is mainly done by the physicians who participated in the short courses or workshops certified by MHME.

### 3.3 The national program for the detection and the prevention of birth defects

There are two recently established comprehensive centers for genetic counseling services in Iran; the second was launched with an academic affiliation in Tehran in 2016 (https://financialtribune.com/articles/people/50834/tehran-s-first-genetic-center-launched). The first one was established by the Shiraz University of Medical Sciences. The genetic counseling services include maternal and pediatric medicine and laboratories of molecular genetics, cytogenetic, and prebirth tests.

Genetic counseling in the area of infertility and abortion has been more highlighted in Iran, and is well established in some special centers which were founded for this specific purpose. Now, there are 2–8 such centers in the capitals/big cities in each province of Iran. The most prominent ones in Tehran (capital) are the infertility clinics at Royan and Avicenna Research Institutes which function as semi‐private and accept the national health insurances. Royan Institute (http://www.royaninstitute.org/)—a leader of stem cell research and also one of the most advanced clinics for infertility treatment in Iran—was established in 1991 as a public nonprofitable organization affiliated to Academic Center for Education, Culture and Research for Reproductive Biomedicine and infertility treatments. Then it this institute was approved by MHME as a cell‐based research center in 1998.

In Tehran, there are some other private clinics of infertility and abortion which are mainly affiliated with the OB/G hospitals or departments. A reason of this progress in terms of quantity and quality of the genetic services in the area of infertility and reproduction could be the government special policies on population growth. Most of these clinics and laboratories are doing PND and PGD (each is part of the IVF division and genetic department) offering molecular and cytogenetic diagnosis mainly for the cases of infertility, recurrent abortion, chromosomal aberration, and sex selection. Prenatal clinics are found in most of them; though a big highly specific center—Hope Generation—for the prenatal clinic including genetics and metabolic genetics exists in Tehran. However, cancer genetics clinics rarely exist; perhaps mainly in MAHAK (https://iscc-charity.org/mahak-charity/) for the malignancies in children.

The last but not the least, most of genetic laboratories in the community are private, although are run by a faculty member of the genetic department. Their main focuses are on the most common monogenic diseases diagnosed via the Sanger sequencing technology, MLPA, Real‐time PCR, RFLP, and FISH.

Premarriage screening and genetic testing has been recently proposed (by scientific and academic Iranian societies since 2016) to be mandatory for all couples in Iran but not approved yet. Upon its approval by the parliament of Iran, the establishment of the genetics comprehensive centers is essential. The mandatory tests include first and second trimester combined screening (Down syndrome, trisomy 13, 18, plasma protein‐A, HCG, and nuchal translucency via ultrasound). Although premarital genetic tests are now mandatory, these limited number of genetic centers may not meet the needs and coverage of the millions of people. There is certainly a real need to expand genetic services in the public health sector in Iran. Financial constraint is currently a serious obstacle for the health sector as well as for the people. On the other hand, Iran still needs the required technologies for some genetic tests; so the samples have to be sent to other countries for standard lab testing.

Screening of congenital hypothyroidism and thalassemia has been successfully performed across the country by the MHME. Apart from thalassemia, a national pilot newborn screening program for other congenital and some frequent genetic diseases/disorders (listed in Table 1) has also been initiated by Iranian health authorities since late 2017. Subjects for the screening of the targeted genetic diseases/disorders (including breast and colon cancers) are the families/individuals with a pattern of heredity.

**Table 1:** National NBS program for congenital and genetic diseases/disorders in Iran

Approved national NBS Program	Pilot national NBS Program (launched since late 2017)
Thalassemia	Down syndrome
Phenylketonuria (PKU)	Hemophilia A—Hemophilia B
	Sickle Cell Disease
	Deafness
	Blindness
	Breast Cancer
	Colon Cancer
	Duchenne & Becker muscular dystrophies
	Mental Retardation
	*Organic‐acid metabolism disorders*
	‐Isovaleric academia (IVA)
	‐Propionic academia (PPA)
	‐Glutaric aciduria (type I)
	‐3‐methyl‐hydroxy Glutaric aciduria
	Multiple carboxylase deficiency
	‐Methyl‐malonic academia (mutase deficiency)
	‐Methyl‐malonic academia (cblA – cblB)
	‐Methylcrotonyl CoA carboxylase (MCC)
	‐Beta‐ketothiolase deficiency
	*Fatty‐acid metabolism disorder*
	‐Medium‐chain acyl‐coA dehydrogenase deficiency (MCADD)
	‐Very long‐chain acyl‐CoA dehydrogenase deficiency (VLCADD)
	Long‐chain 3‐hydroxyacyl‐CoA dehydrogenase (LCHAD) deficiency
	Trifunctional protein deficiency L‐Carnitine absorption problems
	*Amino acid metabolism disorders* Maple Syrup Urine Disease (MSUD) Homocystinuria Citrullinemia Argininosuccinic aciduria Tyrosinemia (type I)

The high cost of genetic services at secondary and tertiary levels does not allow many people to get access to these services despite their needs. Governments will therefore need to allocate necessary resources to make the essential genetic services available for everyone needing these in the community. By considering all shortages in human and financial resources, it is not possible to launch highly special genetic counseling centers in all provinces promptly. However, a 5‐year goal is to set up at least 8–9 such comprehensive genetic centers at prominent medical universities like Isfahan, Tabriz, Mashhad, and Ahvaz, and refer samples from across the country to these laboratories and centers.

### 3.4 Lagging in Genetic Services in Iran

Iran obviously lags behind many developed countries in genetic services, and more diagnostic laboratories are required to make up for such shortcomings. A data network linking all state and private labs is underway in Iran to prevent repetitive and unneeded genetic tests.

Another measure under preparation is clinical guidebooks for development of genetic services and to set the standards for the services. A significant number of samples are sent abroad for genetic diagnostic tests, in a controversial manner between the physicians, patients, MHME, and insurance companies. The Health Reform Plan launched in May 2014 resulted in the establishment of 800 specialized and superspecialty clinics by 2017; 17,000 doctors and specialists are working in these clinics, 10,000 of whom are full‐time staff in the public health sector. However, there is an argument and criticism for no planning of the medical genetics residency programs and disciplines in health education, as well as genetic services, by MHME in the past 3–4 decades.

### 3.5 Current status of genetic diseases in Iran

There is no survey to measure the frequencies and rates of various genetic diseases in the Iranian population and/or in different ethnic subgroups of it. However, based on a few small cohort studies of autosomal recessive and some X‐linked ones, a relatively high‐frequency rate might be possibly found in a single community due to a unique founder mutation. Also in some diseases, the same mutations might be found in several communities either since they were ancient mutations or exchanged by the marriages between them. Lots of (rare) autosomal recessive genetic disease are predicted to be more frequent in Iranian population versus European and American populations. This might be due to high rate of consanguinity, and close/common origin with Caucasians (as some Caucasian countries were) separated from Iran by Russian Empire in the 19th century.

In the past decades, some reports have been published on ethnic‐specific differential pattern and frequencies of mutations in several rare Mendelian diseases in the Iranian population and ethnic groups:

1. Iranian Jews in Israel had a high frequency of inbreeding with very unique and ancient Jewish community and a novel genotype in blood markers (Cohen, Simhai, Steinberg, & Levene, 1981).
2. A particular high frequency of MSI‐H has been reported in sporadic colorectal cancer in Iran (Moghbeli et al., 2011).
3. The relative quantification (RQ) in normal individuals were within the carrier range of 0.31–0.57 for *SMN1* (in SMA), estimating a carrier frequency of 5% in the Iranian population. It is higher than in the European population (Hasanzad et al., 2010).
4. A high prevalence (39.3%) of subjects (respectively, 24.3% homozygous T/T CYP2D6*10 and 15% heterozygous C/T CYP2D6*10 as poor and intermediate metabolizers) among Iranians; therefore, the harmful effects of drugs are relatively common (Bagheri et al., 2015).
5. An *ABCD1* mutation (c.253dup leading to p.Arg85Profs*110) was identified in 35 affected individuals (out of 96 pedigree members) among an expanded pedigree among Lurs. Therefore, the prevalence of X‐ALD is expected to be higher among consanguineous Lurs ethnicity (Mehrpour et al., 2016).
6. )*MSH3, MSH6, APC,* and *PIK3CA* genes are predicted to play a bigger role in the pathogenesis of colon cancer in Iranian population (Ashktorab et al., 2017).
7. In a pilot study of Persian nephropathic cystinosis population, the common 57‐kb deletion was not observed; however, at least 50% of mutations were observed in exons 6 and 7 of *CTNS* (Ghazi et al., 2017).
8. In another pilot study, the role of kidney anion exchanger 1 gene *(AE1)* mutations is highlighted in Iranian children with distal renal tubular acidosis (dRTA); moreover a novel mutation pattern of *AE)* has been reported in patients with distal renal tubular acidosis in Iran (Hooman et al., 2015).
9. Among all Iranian patients with cystic fibrosis, just 16.6% has shown a delta F508 mutation in *CFTR* (either homozygote or heterozygote) (Najafi et al., 2015), which is different from many other populations around the world.

Some of the common genetic disorders which are known as more frequent ones among Iranians include thalassemia, G6PD deficiency, SMA, cystic fibrosis (CF), Familial Mediterranean Fever (FMF), phenylketonuria (PKU), and maple syrup urine disease (MSUD). FMF caused by mutations in the *MEFV* gene has been already reported with a very high prevalence in Middle East Arab, Turkish, Jewish, and Armenian populations. In the recent years, a high 27.4%–25.5% average frequency rate of *MEFV* mutation carriers have been reported among healthy Iranian Azeri Turkish and overall Iranian populations (Bonyadi et al., 2009) Meanwhile, *MEFV* mutations are generally frequent in healthy Iranian individuals across different ethnic groups with the highest rates among Balochs, Gilaks, and Azeri Turkish (Beheshtian et al., 2016) Therefore, an extra screening program for carrier detection in different ethnic groups of Iranians has been highly suggested.

The genetic diseases with a higher referral rate to the author's genetics laboratory at a university hospital in Iran are listed in Table 2.

**Table 2:** The first 25 (monogenic) clinical entities with a higher referral rate in Iran (Based on the author's referral genetics laboratory at the university hospital)

1	B‐thalassemia	*HBH*
2	Thrombophilia	*MTHFR, PAI1*
3	Familial Mediterranean Fever (FMF)	*MEFV*
4	Phenylketunuria (PKU)	*PAH*
5	Cystic Fibrosis (CF)	*CFTR*
6	Maple Syrup Urine Disease (MSUD)	*BCKDHA‐B, DBT*
7	Spinal Muscular Atrophy (SMA)	*SMN1*
8	Fragile X Syndrome	*FMR1*
9	Condenital Adrenial Hyperplasia (CAH) (21‐OH Def.)	*CYP21A2*
10	Primary Hyperoxaluria I	*AGXT1, GRHPR, HOGA1*
11	Cystinosis	*CTNS*
12	Gaucher Disease	*GBA1*
13	Retinoblastoma	*RB1*
14	NBIA	*PANK2*
	PLAN	*PLA2G6*
	Mitochondrial Neurodegeneration	*C19ORF12*
15	Distal RTA	*SLC4A1, ATP6V1B1 ATP6V0A4*
16	Bartter's Syndrome	*SLC12A3, ROMK, CLCNK*
17	Citrillunemia	*ASS1*
18	ADPKD	*PKD1 & PKD2*
19	X‐linked Adrenoleukodystrophy	*ABCD1*
20	Epidermolysis Bullosa	
21	Alport Syndrome	*Col4A*
22	GM1	*GLB1*
23	Ichthyosis	
24	Thyrosinemia I	*FAH*
25	Galactosemia	*GALT*

Congenital defects registries have been recently implemented in Iran; thus there is still no big cohort study for the rates and numbers of Mendelian disorders. However, there is an estimate of significantly higher frequencies for autosomal recessive disorders due to high rate of consanguinity and intraethnic marriages, and close ethnicity with Caucasians in north, northwest, and northeast of Iran (Afzal, Lund, & Skovby, 2018; Keyfi et al., 2018).

Traditionally, a world esophageal cancer belt –with the same or close ethnicity—has been recognized which extends from western/northern China through Mongolia, and the southern part of the former Soviet Union (Kazakhstan, Turkmenistan, Uzbekistan, Tajikistan), Iran, Iraq, and eastern Turkey (Reed & Johnston, 1993). In this regard, a successful 5‐year survey (2004–2008) was performed (as part of GEMINI and Golestan Cohort Study) by the Digestive Disease Research Center (DDRC) of Tehran University of Medical Sciences in collaboration with IARC and the US National Cancer Institute (NCI, NIH). In this study, 50,000 cases were registered in Golestan province which is located in a high‐risk region for esophageal cancer in northern Iran (Pourshams et al., 2010). However, no gene or genetic marker has been yet identified for esophageal cancer in this big cohort.

The Rare Diseases Foundation of Iran (RADOIR) (with an affiliation to “Rare Diseases Patients without Borders”) was established in 2008 to create a patient registry and to help individuals with rate genetic diseases in Iran (http://radoir.com/).

## 4 CONCLUDING REMARKS

Attention has been focused on the field of genetics and genomics in Iran in recent years and some efforts have been enforced and implemented. However, they are totally not adequate, considering the advances in medical genetics and genomics in the past two decades around the world.

Premarital genetic testing in consanguineous and/or intraethnical marriages is highly recommended in Iran and the communities with such population demographics.

Overall, considering the lack of medical genetics residency programs in the Iranian health education system, big demand due to high consanguinity and intraethnic marriages, and the mandatory program of the 5‐year economic development plan, there is a lag in genetic services and necessity to an immediate response to fill this big gap in Iran. Further programs of population carrier detection and prenatal testing for most of monogenic diseases should be implemented because of the high rate of carriers in Iranian population.

The role which NGS technologies play in practicing medical/clinical genetics, disease diagnosis and thus patient management, is prominent and significant. It can also be cost‐effective for a National Health System. However, NGS and the highest quality services in genetics and genomic medicine have not been practically implemented at the clinical level in Iran yet. This pitfall is multifactorial including underestimating the medical/clinical genetics discipline as a clinical residency program by MHME and IRIMC, the recent economic decline in addition to the international and US sanctions against Iran, and some defects in national regulations and administrations. Although as clarified in the National constitution fundamental law and re‐emphasized in the 6th National Development Plan, the Iranian government authority is in charge of providing the standard level of health including genetic services to all Iranian individuals who are in need. The Office of Foreign Assets Control (OFAC) *authorizes the exportation or reexportation to Iran of all items meeting the definition of the term “medical device”* [section 560.530(e)(3) of the ITSR] (https://www.treasury.gov/resource-center/faqs/Sanctions/Pages/faq_iran.aspx#lic_agmed_amend). This includes the NGS equipment and tools; however, the sanctions have a significant negative impact on it indirectly. A considerable part of the delay and lagging in genetic services in Iran, in particular in the recent two decades, has been due to the conflict of interests. Although it was due to the shortage in qualified professional human resources in the field of clinical diagnostic genetic laboratories, a number of the private genetic laboratory directors had been the members of the Iranian Board of Medical Genetics at MHME, while simultaneously serving as advisor/counselor to MHME policy maker(s) in the field of genetics. Synchronously, some of them had also been the members of a team who approved the permission and licenses of other private genetic laboratories, and also in charge of reviewing the complaint files against a laboratory geneticist colleague (as referees). All above‐mentioned parameters and factors resulted in a delay and restriction/constriction in the expansion of services in genetics and genomic medicine in Iran, despite a highly consanguineous population, and despite the big efforts which have been done by a number of medical laboratory geneticists.

## CONFLICTS OF INTERESTS

None declared.
